# Altered ratio of circulating follicular regulatory T cells and follicular helper T cells during primary EBV infection

**DOI:** 10.1007/s10238-020-00621-8

**Published:** 2020-03-23

**Authors:** Jiang Qian, Qinhua Yu, Guoqing Chen, Mingxia Wang, Zhao Zhao, Yueyue Zhang, Liannv Qiu

**Affiliations:** 1 College of Medical Technology, Zhejiang Chinese Medical University, Hangzhou, China; 2 Department of Clinical Laboratory, Zhejiang Provincial People’s Hospital, People’s Hospital of Hangzhou Medical College, 158 Shangtang Road, Hangzhou, 310014 China; 3 Department of Pediatrics, Zhejiang Provincial People’s Hospital, People’s Hospital of Hangzhou Medical College, Hangzhou, China; 4 School of Laboratory Medicine and Life Science, Wenzhou Medical University, Wenzhou, China

**Keywords:** Infectious mononucleosis, Epstein–Barr virus, Follicular regulatory T cells, Follicular helper T cells, Flow cytometry

## Abstract

Follicular help T cells (Tfh) play an important role in the activation and differentiation of B cells, while follicular regulatory T cells (Tfr) control Tfh and resulting humoral immune responses. Accumulating evidence has demonstrated that the dysregulation of Tfr contributed to the pathogenesis of infectious diseases. However, the role of Tfr in Epstein–Barr virus (EBV) infection remains lacking. Fifty-five EBV-infected infectious mononucleosis (IM) patients and 21 healthy individuals (HIs) were recruited in the study. We investigated the number of Tfr (FoxP3^+^CXCR5^+^PD-1^+^CD4^+^) and Tfh (FoxP3^−^CXCR5^+^PD-1^+^CD4^+^) of peripheral blood in IM patients at diagnosis (D0) and day 15 after diagnosis (D15) via multicolor flow cytometry. Results revealed that circulating Tfh (cTfh) and Tfr (cTfr) of IM at D0 were both increased compared to HIs, and cTfr began to decline and return to normal at D15, while cTfh was still higher than those of HIs. More interestingly, the cTfr/cTfh ratio of IM at D0 and D15 was lower than that of HIs, suggesting that the balance between cTfh and cTfr was disturbed during primary EBV infection. Correlation analysis showed a positive correlation between cTfr with CD19^+^IgD^+^CD27^−^ naive B cells, CD19^+^IgD^−^CD27^hi^ plasmablasts or CD19^+^CD24^hi^CD27^hi^ B cells. Moreover, both cTfr and the cTfr/cTfh ratio of IM at D0 were negatively correlated with EBV DNA virus load. These results indicate that an imbalance of cTfr and cTfh cells may be involved in the immunopathogenesis of EBV-infected IM patients and may provide novel strategies for controlling EBV-related disease.

**Electronic supplementary material:**

The online version of this article (10.1007/s10238-020-00621-8) contains supplementary material, which is available to authorized users.

## Introduction

Epstein–Barr virus (EBV) preferentially infects naïve B cells at oropharyngeal lymphoid tissues and subsequently establishes a persistent infection in the circulating memory B cells [1–5]. EBV is strongly associated with nasopharyngeal carcinoma, lymphoma and autoimmune diseases [6–8]. Many T cell subsets and NK are suggested to be involved in immune response to EBV infection. Nevertheless, the mechanisms the human immune system in the control of EBV infection is not completely understood. EBV-infected infectious mononucleosis (IM) patients provide an excellent model for the study of immune responses against EBV.

Follicular helper T cells (Tfh), a novel subset of CD4^+^ T helper cells, are essential for the formation of germinal centers (GCs) and humoral response [9]. Tfh bind to B cells via CXCR5 on the surface of Tfh and its ligand CXCL13 in the follicular GCs of B cells, then migrate to B cell follicles/GCs in the lymph nodes and support B cell activation, expansion and differentiation into plasma cells and generate B-cell memory in GCs [9]. In contrast, follicular regulatory T cells (Tfr) are an effector subset of regulatory T cells (Treg), which control GCs cell number and Tfh function but have varied effects on the quality and antigen specificity of the response [10–14]. Studies suggest that partial or temporary disruption of Tfr function leads to increased availability of costimulatory molecules and increase in the antigen-specific immune response [15–18]. Accumulating evidence has demonstrated that circulating Tfr (cTfr) and Tfh (cTfh) have been found in blood and display phenotypic and functional feature of classical Tfr and Tfh [19–21]. Recent studies revealed that cTfr expand during chronicviral and parasitic infections such as human immunodeficiency virus (HIV), hepatitis B virus (HBV), hepatitis C virus (HCV), and Schistosoma japonica [22–24]. Increased cTfr frequency in patients with chronic HBV or HCV was strongly associated with serum viral load of both infections. Miles et al. [24] found Tfr contribute to inefficient GCs responses and inhibit HIV and SIV clearance. Botta et al. [25] found that Tfr were elevated in the late immune response to influenza infection and inhibited the production of autoimmune antibodies. Moreover, Dhaeze et al. [26] showed that cTfr increased after influenza vaccination and were correlated with anti-flu Ab responses. An altered balance of cTfh and cTfr has been associated with autoimmune diseases such as systemic lupus erythematosus [27], rheumatoid arthritis [28].

To date, few studies have focused on the role of cTfr during EBV infection. Our previous study found that cTfh cells were high in IM patients, which was negatively correlated with naive B cells and positively correlated with memory B cells and plasmablasts [29]. How cTfr arise and evolve over time during EBV infection and the role of cTfr and the effect of cTfr and cTfh balance on B cell differentiation during primary EBV infection remain largely unknown. Here, we observed the balance of cTfr and cTfh and the association cTfr or the cTfr/cTfh ratio with different B subsets in primary EBV-infected IM patients.

## Materials and methods

### Subjects

Fifty-five IM patients and 21 healthy individuals (HIs) from Zhejiang Provincial People’s Hospital of Hangzhou Medical College were recruited in this study. Serological screening was performed to confirm their EBV infection and to exclude other virus or bacteria infection, including herpes simplex virus 2, rubella virus, cytomegalovirus, toxoplasma, rotavirus, coxsackie virus, mycoplasma, chlamydia, and hepatitis A, B, C and D. Patients with irrelevant chronic diseases or other autoimmune diseases were excluded. Clinical characteristics of the IM patients and HIs are shown in Table 1. EDTA-K_2_ anticoagulated peripheral blood samples were collected at diagnosis (D0) and day 15 after diagnosis (D15) and detected within 24 h. Informed parental written consent was obtained in accordance with the Ethics Committee of Zhejiang Provincial People’s Hospital and the Declaration of Helsinki.

**Table 1:** Clinical characteristics of IM patients and healthy individuals

	HIs	IM	*P* value
Sex	21	55	0.709
Male	12	34	
Female	9	21	
Age(years)	6.33 ± 4.26	4.72 ± 2.87	0.066
WBC (*10^9^/L)	8.31 ± 2.42	13.13 ± 5.32	0.0002
LYM (*10^9^/L)	3.07 ± 1.11	8.15 ± 4.48	< 0.0010
EBV DNA (copies/mL)	–	256740.5 ± 853540.2	0.000
EB-VCA-IgM (IU/L)	–	11.99 ± 5.86	0.000

*WBC* white blood cell, *LYM* lymphocyte, *EBV DNA* Epstein–Barr virus deoxyribonucleic acid, *EB-VCA-IgM* Epstein–Barr viral capsid antigens immunoglobulin M

Analysis of *T* test, Chi-squared test; *P* < 0.05 was considered statistically significant

### Flow cytometry

Peripheral blood mononuclear cells (PBMCs) were acquired by Ficoll-Hypaque density gradient separation (Dakewei, China) and then were stained for the following surface markers of cTfr and cTfh for 20 min: CD4-PC7 (Clone 13B8.2, Beckman Coulter), CXCR5-Alexa Fluor^®^488 (Clone RF8B2, BD Biosciences) and PD-1-PerCP-Cy5.5 (Clone EH12.1, BD Biosciences). After surface marker staining, cells were fixed and permeabilized using a Cytofix/Cytoperm kit (BD Biosciences, San Diego, CA, USA) and then incubated intracellularly with FoxP3-PE (Clone 259D/C7, BD Biosciences) for 30 min. Cells were washed and were acquired on FACSCano™II flow cytometer, and data were analyzed by FACSDiva software (BD Biosciences, USA). Isotype-matched antibodies were used in all procedures. The strategy is shown in Supplementary Figure 1. FoxP3^+^CXCR5^+^PD-1^+^CD4^+^ cells were defined as Tfr, whereas FoxP3^−^CXCR5^+^PD-1^+^CD4^+^ were as Tfh. The CD4^+^ T cell absolute number of each patient in peripheral blood was obtained by peripheral blood routine lymphocyte subsets analysis and the use of fluorescent bead system. The absolute number of Tfr and Tfh was multiplied by the absolute number of CD4^+^ T cells and the corresponding percent of Tfr and Tfh. PBMCs were stained for the following surface markers to characterize B cell subsets as following: IgD-APC (Clone 11-26c, BD Biosciences), CD24-PerCP-Cy5.5 (Clone ML5, BD Biosciences), CD27-PE (Clone L128, BD Biosciences) and CD19-FITC (Clone J4.119, Beckman Coulter). The strategy of different B cells is shown in Supplementary Figure 2. CD19^+^IgD^+^CD27^−^ cells were defined as naive B cells, CD19^+^IgD^+^CD27^+^ cells were as memory B cells, while CD19^+^IgD^−^CD27^hi^ cells were as plasmablasts.

### EBV serology and EBV DNA quantification

Serum samples were measured immunoglobulin (Ig) M (IgM) viral capsid antigen (VCA) using enzyme immunoassays (Liason VCA IgM, DiaSorin S.p.A.) according to the manufacturer’s instructions. EBV DNA was measured using a commercial real-time PCR kit, amplifying a 191 bp region of the EBNA-1 gene (BioQuant EBV, Biodiversity), according to the manufacturer’s protocol, and detected by the ABI PRISM 7500 Sequencer Detection System (Applied Biosystems, USA).

### Statistical analysis

Statistical analysis was performed with GraphPad Prism 5.01 software. The quantitative data were presented as the mean values ± standard deviations. Statistical tests for data analysis included one-way ANOVA test and Spearman r correlation. *P* value < 0.05 were considered to be statistically significant.

## Results

### cTfr and cTfh were significantly increased, while the cTfr/cTfh ratio was significantly decreased in IM patients

**Fig. 1:**
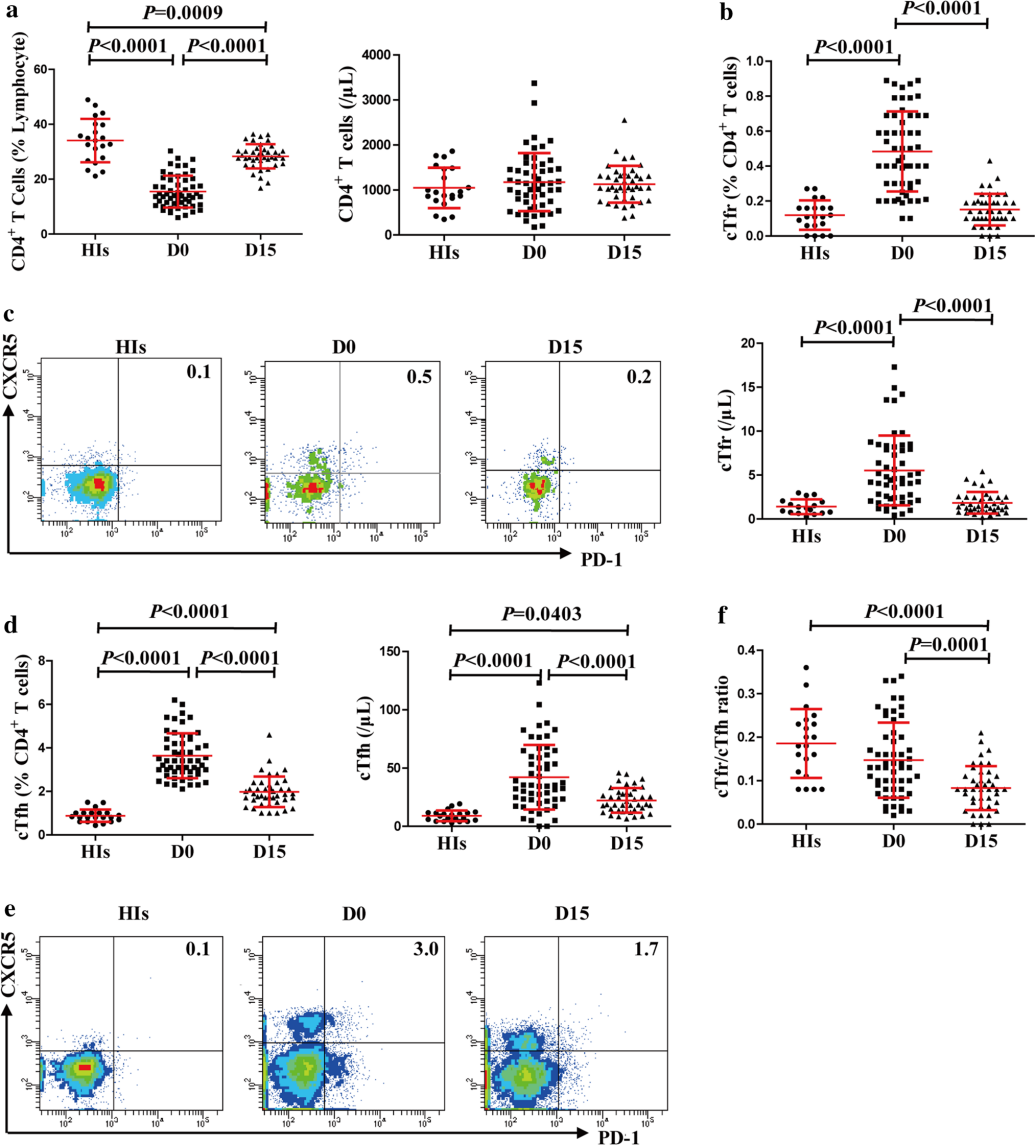
Elevated levels of cTfr and cTfh but low cTfr/cTfh ratio in IM patients. **a** Frequency in lymphocytes (left) and the number (right) of CD4^+^ T cells among HIs (*n* = 21) and IM patients (*n* = 55) at D0 and D15, **b** frequency in CD4^+^ T cells (top) and the number of cTfr cells (bottom) from the peripheral blood of HIs and IM patients at D0 and D15, **c** representative flow cytometric dot plots of cTfr on CD4^+^FoxP3^+^ T cells, **d** frequency in CD4^+^ T cells (left) and the number (right) of cTfh from the peripheral blood of HIs and IM patients at D0 and D15, **e** representative flow cytometric dot plots of cTfh on CD4^+^ FoxP3^−^ T cells, **f** the cTfr/cTfh ratio in HIs and IM patients at D0 and D15. D0: at diagnosis; D15: day 15 after diagnosis

The number of CD4^+^ T cells of IM patients was no difference from that of HIs, though the frequency of CD4^+^ T cells was significantly decreased in IM patients compared to HIs (Fig. 1a). Besides increased levels of cTfh cells, we also observed a significant increase in the cTfr cells during acute EBV infection (D0) in IM compared to HIs (Fig. 1b, c). Then, we observed that both cTfr and cTfh began to decline and were significantly lower at D15 than those at D0, but Tfh at D15 were still higher than HIs (Fig. 1b, d). More importantly, when we further analyzed the cTfr/cTfh ratio, the results showed that cTfr/cTfh ratio at D15 is significantly lower than HIs, and cTfr/cTfh ratio at D0 is low compared with HIs but had no statistical significance (Fig. 1f). These data suggested the imbalance between cTfr and cTfh during acute EBV infection.

### Positive correlation between cTfr and CD19^+^IgD^+^CD27^−^ naive B cells, CD19^+^IgD^−^CD27^hi^ plasmablasts and CD19^+^CD24^hi^CD27^hi^ B cells

**Fig. 2:**
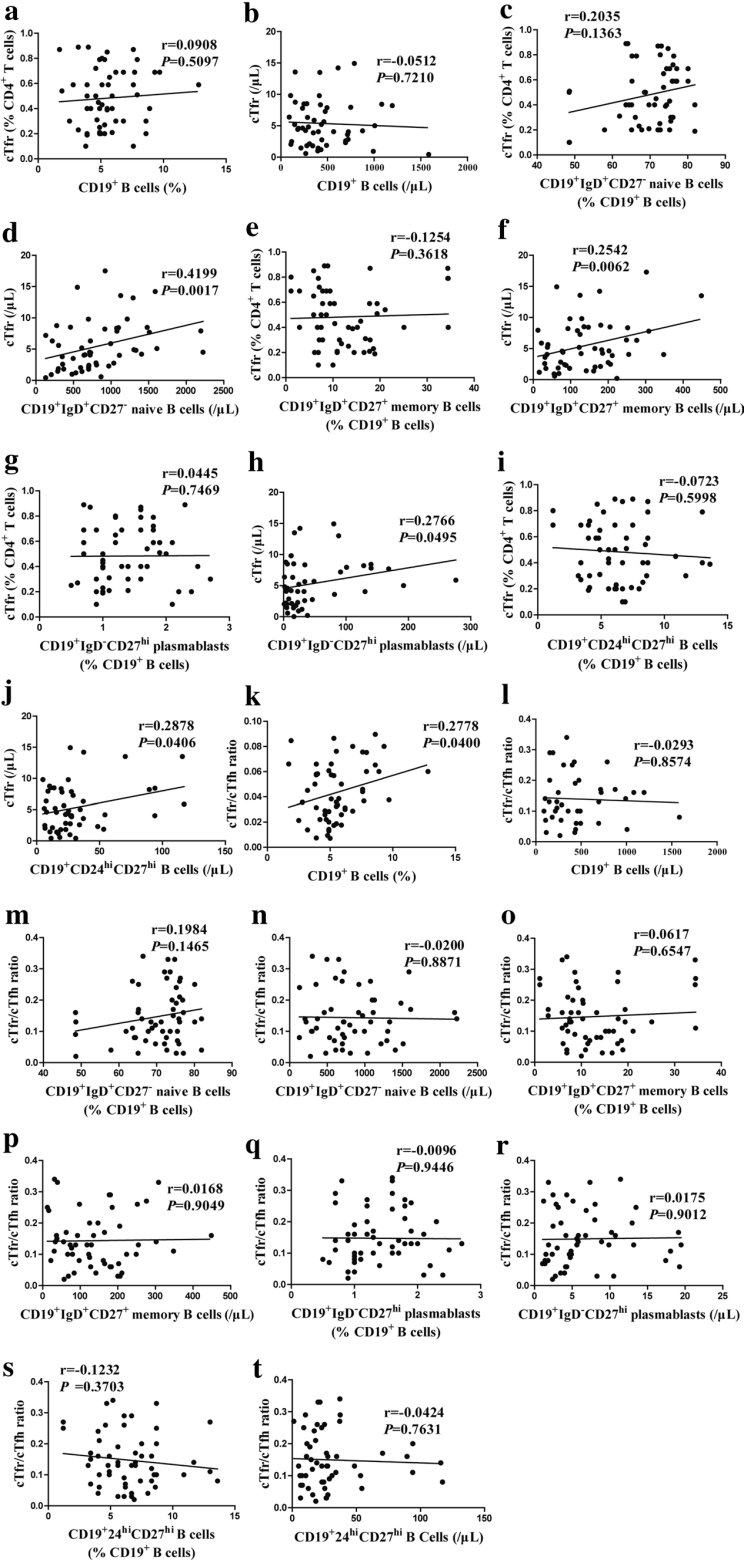
Positive correlation between cTfr and CD19^+^IgD^+^CD27^−^ naive B cells, CD19^+^IgD^−^CD27^hi^ plasmablasts or CD19^+^CD24^hi^CD27^hi^ B cells in IM patients at D0. Correlation between the frequency and number of cTfr and CD19^+^ B cells (**a**, **b**), CD19^+^IgD^+^CD27^−^ naive B cells (**c**, **d**), CD19^+^IgD^+^CD27^+^ memory B cells (**e**, **f**), CD19^+^IgD^−^CD27^hi^ plasmablasts (**g**, **h**), CD19^+^CD24^hi^CD27^hi^ B cells (**i**, **j**); correlation between the cTfr/cTfh ratio and frequency and number of CD19^+^ B cells (**k**, **l**), CD19^+^IgD^+^CD27^−^ naive B cells (**m**, **n**), CD19^+^IgD^+^CD27^+^ memory B cells (**o**, **p**), CD19^+^IgD^−^CD27^hi^ plasmablasts (**q**, **r**), CD19^+^CD24^hi^CD27^hi^ B cells (**s**, **t**)

B cells play a central role in the pathogenesis of infectious disease. CD19^+^CD24^hi^CD27^hi^ B cells have been demonstrated to downregulate inflammatory reactions and induce tolerance by production of IL-10 and/or TGF-*β* [30, 31]. Our previous study found cTfh were negatively correlated with naïve B cells and positively correlated with memory B cells and plasmablasts. Whether increased cTfr or low cTfr/cTfh ratio will affect the B cell differentiation during primary EBV infection is unknown. Herein, we analyzed the correlation between cTfr or the cTfr/cTfh ratio and different B cell subsets, which included CD19^+^ B cells, CD19^+^IgD^+^CD27^−^ naïve B cells, CD19^+^IgD^+^CD27^+^ memory B cells, CD19^+^IgD^−^CD27^hi^ plasmablasts, CD19^+^CD24^hi^CD27^hi^ B cells. Our data showed a positive correlation between cTfr and CD19^+^IgD^+^CD27^−^ naive cells, CD19^+^IgD^−^CD27^hi^ plasmablasts or CD19^+^CD24^hi^CD27^hi^ B cells (Fig. 2d, h, j). However, no correlation between the cTfr/cTfh ratio and these B cell subsets was found (Fig. 2k–t).

### Upregulation of cTfr was negatively correlated with EBV DNA viral load in IM patients

**Fig. 3:**
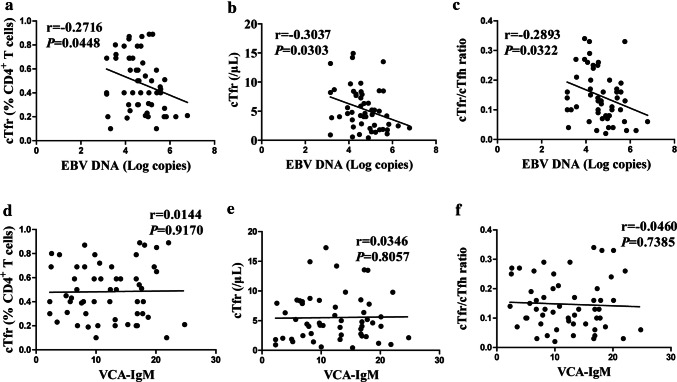
Upregulation of cTfr was negatively correlated with the EBV DNA viral load in IM patients at D0. Correlation between the frequency (**a**) and number (**b**) of cTfr and EBV DNA viral load. Correlation between the cTfr/cTfh ratio and EBV DNA viral load (**c**). Correlation between the frequency (**d**) and number (**e**) of cTfr cells and EB-VCA-IgM. Correlation between the cTfr/cTfh ratio and EB-VCA-IgM (**f**)

cTfr were increased, but the cTfr/cTfh ratio was decreased during primary EBV infection, which prompts us to wonder whether cTfr or the cTfr/cTfh ratio is related to EBV DNA viral load during primary EBV infection. Correlation analysis showed a significant negative correlation between cTfr or the cTfr/cTfh ratio and EBV DNA viral load (Fig. 3a–c**).** However, we found no correlation between cTfr or the cTfr/cTfh ratio and EB-VCA-IgM in IM patients (Fig. 3d–f).

## Discussion

Tfh are important for helping B cell activation and differentiation, while Tfr suppress B cell responses through modulation of Tfh and GCs development [6]. However, Laidlaw et al. [32] observed high IL-10-producing Tfr to support GCs responses in mouse models of acute lymphocytic choriomeningitis virus infection. Our findings extend the previous observations of a high Tfh in IM patients [29]. In this study, we also found a significant increase in cTfr in IM patients during primary EBV infection, indicating that an increased cTfr may be a marker of ongoing humoral activity, similar to other study [27, 28, 32]. Most importantly, we found that cTfr were positively correlated with CD19^+^IgD^+^CD27^−^ naive B cell and CD19^+^IgD^−^CD27^hi^ plasmablasts. Therefore, in this observation study, these finding suggest high cTfr may inhibit B cell differentiation in the immune response to primary EBV infection. Further study is needed to investigate the function of this elevated Tfr.

CD19^+^CD24^hi^CD27^hi^ B cells, a distinct subset of regulatory B cells (Bregs), could be potent suppressors of immunity which can suppress proinflammatory Th1/Th17 responses and induce Treg by production of IL-10 and/or TGF-*β* [33]. Li et al. [34] found that IL-10^+^ regulatory B cells were significantly higher following incubation with Tfr than with non-Tfr Treg, which suggested that Tfr were more potent at inducing IL-10^+^ regulatory B cells. Siewe et al. [35] found that high in IL-10-producing CD19^+^CD24^hi^CD27^hi^ B cells had been shown to be positively correlated with HIV viral load in HIV-infected patients. Our data showed a positive correlation between cTfr and CD19^+^CD24^hi^CD27^hi^ B cells. Most interesting, we found a negative correlation between cTfr or the cTfr/cTfh ratio and EBV DNA load. These results suggest that cTfr may induce the production of CD19^+^CD24^hi^CD27^hi^ B cells, further lead to immune dysfunction during primary EBV infection and hinder EBV clearance.

Given that Tfh and Tfr are reciprocal and antagonistic regulators of GCs responses, a balance of Tfh and Tfr is critical for immune homeostasis. A disordered cTfr/cTfh ratio is associated with the development of infectious disease [12]. In this study, we found significantly low cTfr/cTfh ratio during primary EBV infection, which indicates an imbalance between cTfr and cTfh during primary EBV infection. During the course of an immune response to primary EBV infection, both cTfh and cTfr begin to proliferate; however, cTfh proliferate faster and skew the proportion in favor of helper capacity. By the D15, cTfr have return to normal, while cTfh proliferation continues, which cause a shift from immune tolerance state to immune responsive.

Our previous study has demonstrated that Tfh cells were positively correlated with EBV DNA load during primary EBV infection [29]. Most interestingly, we here also observed a negative correlation between cTfr or the cTfr/cTfh ratio and EBV DNA viral load, which indicated that the viral control was probably hindered by the development of immunosuppressive Tfr cells. Therefore, we think the balance of cTfr and cTfh cells may play a role in controlling EBV and cTfr may play a role in controlling the Tfh response and limiting the spontaneous immune damage induced by excessive immunity during primary EBV infection. Our data showed a positive correlation between cTfr and CD19^+^IgD^+^CD27^−^ naive B cell, CD19^+^IgD^−^CD27^hi^ plasmablasts. These results suggest that Tfr contribute to inefficient GCs responses and hinder EBV clearance, similar to the observation of Miles et al. [24].

Taken together, our study indicates that low cTfr/cTfh ratio may contribute to immunopathogenesis in the immune response to EBV infection and hinder EBV clearance. These results provide unique insight into the underlying immune response of primary EBV infection and suggest potential strategies for controlling EBV-related disease through suppressing cTfr.

## Electronic supplementary material

Below is the link to the electronic supplementary material.

Supplementary Figure 1 Gating strategy applied to identify Tfr and Tfh. Tfh were pre-gated on CD4^+^FoxP3^-^ T cells and examined for the levels of CXCR5 and PD-1. Tfr were pre-gated on CD4^+^FoxP3^+^ T cells and examined for the levels of CXCR5 and PD-1 (TIFF 8045 kb)

Supplementary Figure 2 Gating strategy applied to identify different B cell subsets. CD19^+^IgD^+^CD27^-^ naive B cells, CD19^+^IgD^+^CD27^+^ memory B cells and CD19^+^IgD^-^CD27^hi^ plasmablasts were pre-gated on CD19^+^ B cells and examined for the levels of IgD and CD27. CD19^+^CD24^hi^CD27^hi^ B cells were pre-gated on CD19^+^ B cells and examined for the levels of CD24 and CD27 (TIFF 8143 kb)
