# Supplementary figures and images for: Altered ratio of circulating follicular regulatory T cells and follicular helper T cells during primary EBV infection
Source: Clin Exp Med. 2020 Mar 23;20(3):373–80. doi: 10.1007/s10238-020-00621-8 (PMC7366583; doi:10.1007/s10238-020-00621-8)

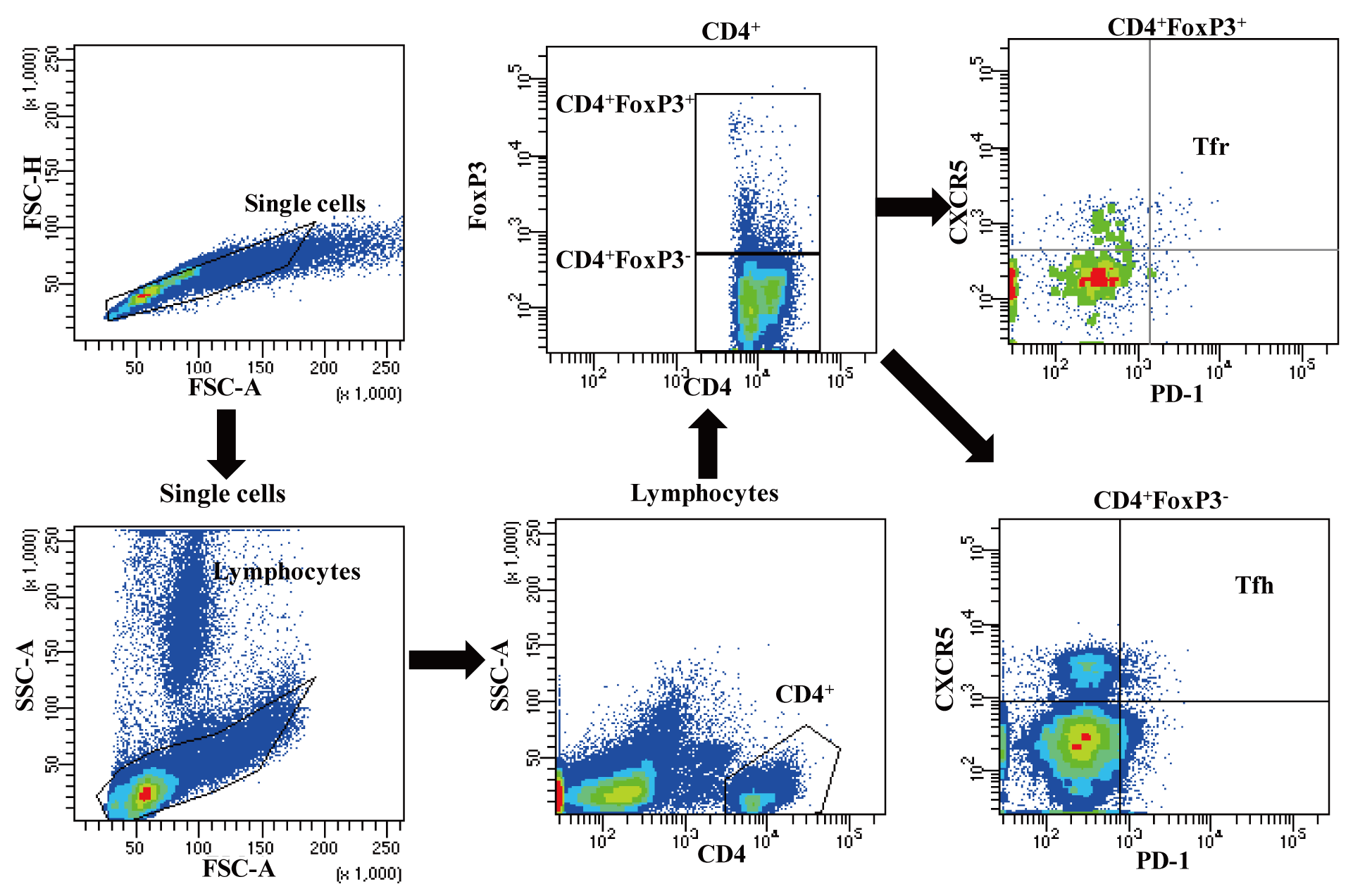

Supplement: Supplementary file 1 — Gating strategy applied to identify Tfr and Tfh. Tfh were pre-gated on CD4+FoxP3- T cells and examined for the levels of CXCR5 and PD-1. Tfr were pre-gated on CD4+FoxP3+ T cells and examined for the levels of CXCR5 and PD-1 (TIFF 8045 kb) [file 10238_2020_621_MOESM1_ESM.tif]

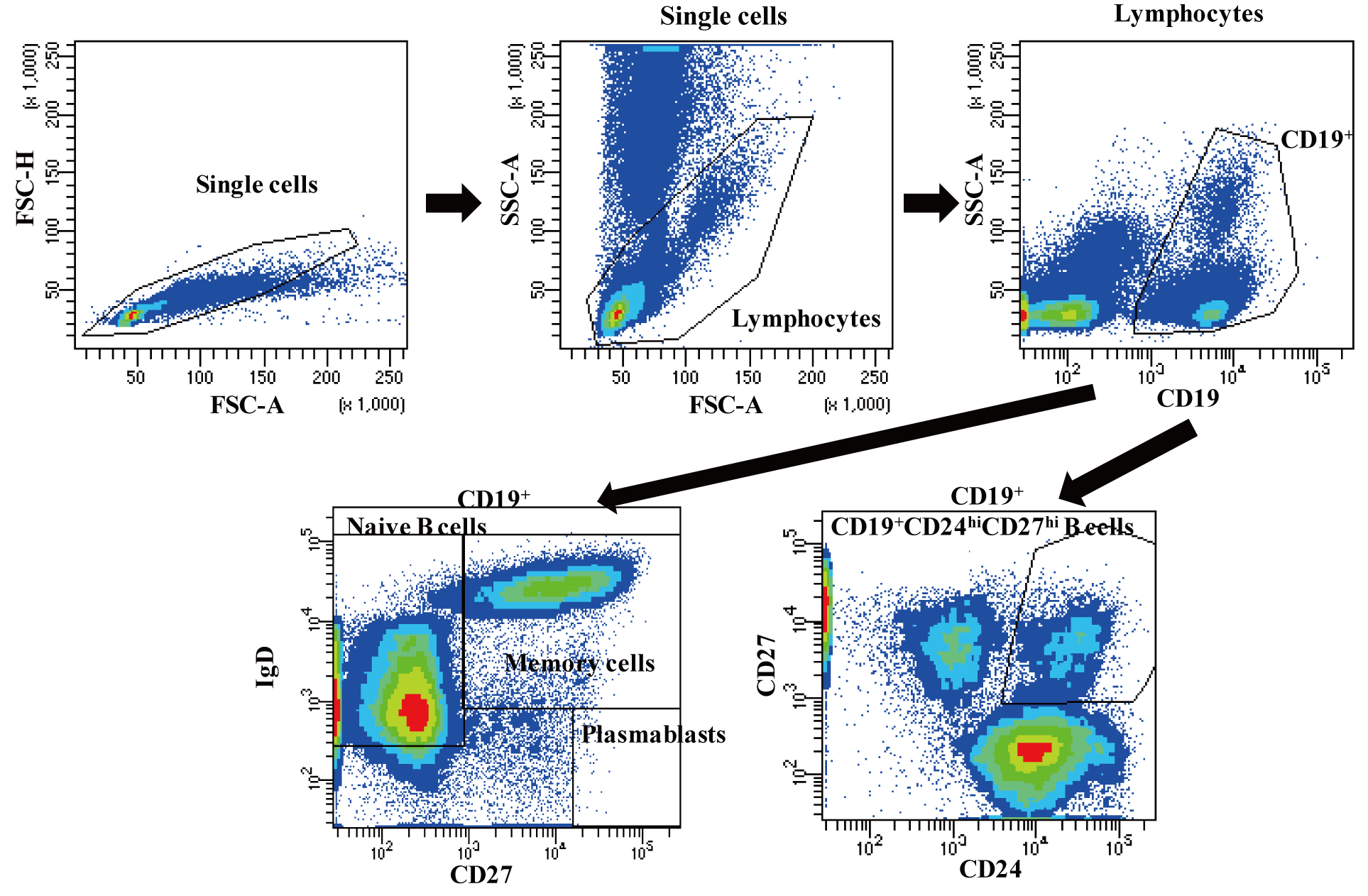

Supplement: Supplementary file 2 — Gating strategy applied to identify different B cell subsets. CD19+IgD+CD27- naive B cells, CD19+IgD+CD27+ memory B cells and CD19+IgD-CD27hi plasmablasts were pre-gated on CD19+ B cells and examined for the levels of IgD and CD27. CD19+CD24hiCD27hi B cells were pre-gated on CD19+ B cells and examined for the levels of CD24 and CD27 (TIFF 8143 kb) [file 10238_2020_621_MOESM2_ESM.tif]
